# Increased frequencies of circulating CXCL10-, CXCL8- and CCL4-producing monocytes and Siglec-3-expressing myeloid dendritic cells in systemic sclerosis patients

**DOI:** 10.1007/s00011-017-1106-7

**Published:** 2017-11-10

**Authors:** Tiago Carvalheiro, Sara Horta, Joel A. G. van Roon, Mariana Santiago, Maria J. Salvador, Hélder Trindade, Timothy R. D. J. Radstake, José A. P. da Silva, Artur Paiva

**Affiliations:** 1 Blood and Transplantation Center of Coimbra, Portuguese Institute of Blood and Transplantation, Coimbra, Portugal; 2 Department of Rheumatology and Clinical Immunology, University Medical Center Utrecht, Utrecht, The Netherlands; 3 Laboratory of Translational Immunology, University Medical Center Utrecht, Utrecht, The Netherlands; 4 Department of Chemistry, University of Aveiro, Aveiro, Portugal; 5 Department of Rheumatology, Coimbra University Hospital Center, Coimbra, Portugal; 6 Faculty of Medicine, University of Coimbra, Coimbra, Portugal; 7 Flow Cytometry Unit, Clinical Pathology Service, Coimbra University Hospital Center, Praceta Prof. Mota Pinto, Ed. S. Jerónimo, 3° piso, 30001-301 Coimbra, Portugal

**Keywords:** Classical monocytes, Non-classical monocytes, Myeloid dendritic cells, Inflammation, Chemokines, Systemic sclerosis

## Abstract

**Objective:**

To investigate the ex vivo pro-inflammatory properties of classical and non-classical monocytes as well as myeloid dendritic cells (mDCs) in systemic sclerosis (SSc) patients.

**Methods:**

Spontaneous production of CXCL10, CCL4, CXCL8 and IL-6 was intracellularly evaluated in classical, non-classical monocytes and Siglec-3-expressing mDCs from peripheral blood of SSc patients and healthy controls (HC) through flow cytometry. In addition, production of these cytokines was determined upon toll-like receptor (TLR) 4 plus Interferon-γ (IFN-γ) stimulation.

**Results:**

The frequency of non-classical monocytes spontaneously producing CXCL10 was increased in both limited (lcSSc) and diffuse cutaneous (dcSSC) subsets of SSc patients and CCL4 was augmented in dcSSc patients. The proportion of CCL4-producing mDCs was also elevated in dcSSc patients and the percentage of mDCS producing CXCL10 only in lcSSc patients. Upon stimulation, the frequency of non-classical monocytes expressing CXCL8 was increased in both patient groups and mDCs expressing CXCL8 only in lcSSc. Moreover, these parameters in unsupervised clustering analysis identify a subset of patients which are characterized by lung fibrosis and reduced pulmonary function.

**Conclusions:**

These data point towards a role of activated non-classical monocytes and mDCs producing enhanced levels of proinflammatory cytokines in SSc, potentially contributing to lung fibrosis.

**Electronic supplementary material:**

The online version of this article (10.1007/s00011-017-1106-7) contains supplementary material, which is available to authorized users.

## Introduction

Systemic sclerosis (SSc) is a chronic connective tissue disease, characterized by vascular abnormalities and immunological disturbances followed by progressive fibrosis of the skin and internal organs. SSc is recognized as the most severe connective tissue disorder and the most resistant to therapy, being associated with the highest case-specific mortality among rheumatic diseases. The etiology of SSc is largely unknown and its pathogenesis is complex and poorly understood [1, 2].

Recently, accumulating evidence underlines the importance of immune cells in SSc pathogenesis [3, 4]. Research on the genetic risk factors for SSc has shown that all susceptibility genes, robustly replicated so far, are immune regulating genes, thus supporting a strong autoimmune component underlying SSc pathogenesis as observed in other autoimmune diseases [4–6]. On the basis of these aforementioned observations, it is now generally accepted that the activation and transformation of fibroblasts is, at least partly, a downstream effect of the perturbed immune response triggered by, e.g. aberrant toll-like receptor (TLR) response and dysregulated interferon (IFN) response [7–9]. This is associated by enhanced secretion of inflammatory mediators such as IFNs and IFN-induced molecules such as Interferon gamma-induced protein 10-IP-10 (CXCL10), and inflammatory chemokines such as CXCL4 [10–12]. In addition, CXCL8 (IL-8), CCL4 (MIP-1β) and IL-6 have been implicated in the disturbed immune activation in SSc patients [13–15]. Despite these observations, identification of the immune cells that contribute to the immunopathology remains largely elusive in SSc. In particular, the contribution of myeloid lineages to the disease is unclear.

At present, it is well accepted that monocytes represent a heterogeneous cell population in the blood, where it is possible to distinguish classical and non-classical monocyte subsets [16]. The heterogeneity of blood monocytes and their previously described functions suggests that each subpopulation could play a different role during homeostasis and in disease conditions [17]. Blood myeloid dendritic cells (mDC) strongly express HLA-DR and the myeloid-associated antigens CD11c and CD33 (Siglec-3) suggesting their direct derivation from the myeloid lineage [16, 18, 19].

Monocytes and DC subsets express high levels of TLRs which are essential to fulfill their role as sentinel cells of the immune system. As such, they might constitute the early TLR-mediated response. It has been shown that TLR4 signalling robustly augments transforming growth factor-β responses, a relevant mechanism for maintaining and amplifying fibrosis in SSc [20]. Furthermore, fibronectin extra domain A (FnEDA), an endogenous TLR4 ligand, is elevated in the circulation and skin lesions of SSc patients and this FnEDA–TLR4 axis is implicated in cutaneous fibrosis [21]. S100A8/A9 is also considered an endogenous ligand for TLR4 and its levels were also found increased in SSc, especially in the limited cutaneous form of the disease, with lung fibrosis [22]. Total monocytes, monocyte-derived DCs and mDCs were found to have an aberrant cytokine response after TLR responses stimulation in SSc [23, 24]. However, subsets of monocytes and mDCs have not been studied ex vivo yet, especially regarding their chemokine production.

Along with the TLR4 dysfunction, there is evidence for a prominent IFN signature in SSc. These genes are recurrently differentially expressed in circulation, but also in lung tissue [11, 25, 26]. Altogether, TLR and IFN signalling appear to play a pivotal role in the onset and/or perpetuation of the fibrotic response observed in SSc and warrants further investigation.

Here, we investigated, ex vivo, to what extent circulating cytokine and chemokine-producing monocytes and dendritic cells might contribute to SSc and assessed whether TLR/IFN-mediated signalling is aberrant in these patients.

## Materials and methods

### Patients

Forty patients fulfilling American College of Rheumatology Criteria for the classification of SSc [27] followed at Rheumatology Department of Coimbra University Hospital Center were recruited and classified according to LeRoy et al. [28] as having limited cutaneous SSc (lSSc, *n* = 27) or diffuse cutaneous SSc (dSSc, *n* = 13) (Table 1). A clinical evaluation was made, including disease duration, modified Rodnan skin score (mRss), digital necrosis and assessment of target organs’ involvement. Organs’ involvement was evaluated according to clinical practice: pulmonary function was determined by spirometry, lung fibrosis by high resolution CT scanning, renal involvement was identified by previous scleroderma renal crisis, and significant increase of creatinine level or proteinuria. The autoantibody profile was collected from medical records (Table 1).

**Table 1:** Demographic and clinical data of healthy controls and patients with systemic sclerosis (SSc)

Characteristics	Healthy controls	Limited cutaneous SSc	Diffuse cutaneous SSc
Number (*n*)	20	27	13
% within SSc group	–	67.5%	35.5%
Female gender [*n* (%)]	16 (80%)	21 (77.8%)	10 (76.9%)
Present age, years (mean ± SD)	52.0 ± 9.9	52.1 ± 13.6	55.9 ± 9.5
Disease duration, years (mean ± SD)	–	9.9 ± 8.5	8.5 ± 9.0
Isolated anti-nuclear antibodies [*n* (%)]	–	9 (33.3%)	0 (0%)
Anti-topoisomerase I antibodies [*n* (%)]	–	0 (0%)	13 (100%)
Anti-centromere antibodies [*n* (%)]	–	18 (66.7%)	0 (0%)
Modified Rodnan skin score (mean ± SD)	–	10.7 ± 6.6	17.1 + 10.7
Pulmonary hypertension [*n* (%)]	–	1 (3.7%)	2 (15.4%)
Lung fibrosis (presence or history) [*n* (%)]	–	8 (29.6%)	8 (61.5%)
Digital ulcers (presence or history) (n (%))	–	9 (33.3%)	7 (53.8%)
Current treatments [*n* (%)]	–		
Vasodilators	–	26 (100%)	13 (100%)
ACE inhibitors (angiotensin-converting enzyme inhibitors)	–	7 (26.9%)	4 (30.8)
Corticoids	–	11 (42.3%)	6 (46.2%)
Immunosuppressives	–	4 (15.4%)	0 (0%)

The healthy control group (HC) consisted of 20 healthy individuals (80% female, 20% male, mean age 52.0 ± 11.7 years). These participants were required to complete a brief questionnaire regarding previous or current medical conditions. All were free from autoimmune disease, active inflammatory conditions or current treatment with any immunomodulatory drugs.

Informed consent was obtained from all individual participants included in the study.

### In vitro stimulation of monocytes subsets and myeloid dendritic cells

Peripheral blood (PB) samples from each individual were collected into heparinized tubes and immediately processed. PB samples (0.5 mL) were diluted (1:1), in RPMI-1640 medium (Life Technologies—Thermo Fisher Scientific; Carlsbad, CA, USA), supplemented with 2 mM L-glutamine and antibiotic–antimycotic agent (Life Technologies—Thermo Fisher Scientific) (in a total of 1 mL) in the presence of 10 μg/mL of Brefeldin A (Sigma, St. Louis, MO, USA) to prevent the release of soluble mediators from the cells. In addition, 100 ng/mL of lipopolysaccharide (LPS) from Escherichia coli (serotype 055:B5; Sigma, St. Louis, MO, USA) plus 100 U/mL of recombinant interferon-γ (IFN-γ) (Becton and Dickinson (BD) Pharmingen, San Diego, CA, USA) were added to one of the tubes (stimulated whole blood) while another tube, with just Brefeldin A, was used for analysis of the spontaneous ex vivo production (unstimulated whole blood). Samples were incubated during 6 h at 37 °C in a 5% CO_2_ and 95% humidity sterile environment.

### Flow cytometry

After this incubation time, samples were aliquoted in different tubes (200 µL per tube) and stained according to a four-color flow cytometry strategy with the Dendritic Cell Exclusion Kit (Cytognos, Salamanca, Spain), a mix of monoclonal antibodies containing anti-CD3, anti-CD19, anti-CD56 and anti-CD14 conjugated with fluorescein isothiocyanate. Additionally, samples were stained with anti-HLA-DR peridinin chlorophyll protein-cyanin 5 (clone: L243; BD Biosciences, San Jose, CA, USA) and anti-CD33 allophycocyanin (clone: P67.6; BD Biosciences).

Cells were then incubated for 15 min at room temperature in darkness. Following this incubation period, cells were fixed, permeabilized, and stained with phycoerythrin-conjugated monoclonal antibodies directed against different intracytoplasmic: CXCL8—IL-8 (clone: AS14; BD Biosciences), CXCL10—IP-10 (clone: 6D4; BD Pharmingen), CCL4—MIP-1β (clone: D21-1351; BD Pharmingen) and IL-6 (clone: MQ2-6A3; BD Pharmingen) using the IntraPrep Permeabilization Reagent (Beckman Coulter—Immunotech; Marseille, France), according to the manufacturer’s recommendations. Cells were resuspended in 0.5 mL of PBS before acquisition in a FACSCalibur flow cytometer (BD Biosciences) equipped with an argon ion laser and a red diode laser using the BD CellQuest application (BD Biosciences). At least 200,000 nucleated events were acquired per sample.

### Flow cytometry data analysis

The flow cytometry data was analyzed with the Infinicyt v1.6 (Cytognos) software. The evaluation of CXCL8, CXCL10, CCL4 and IL-6 production was based on the frequency (%) of positive cells within each cell subset (Fig. S1). Isotype-matched negative controls were used to establish cut-off values for positivity.

**Fig. 1:**
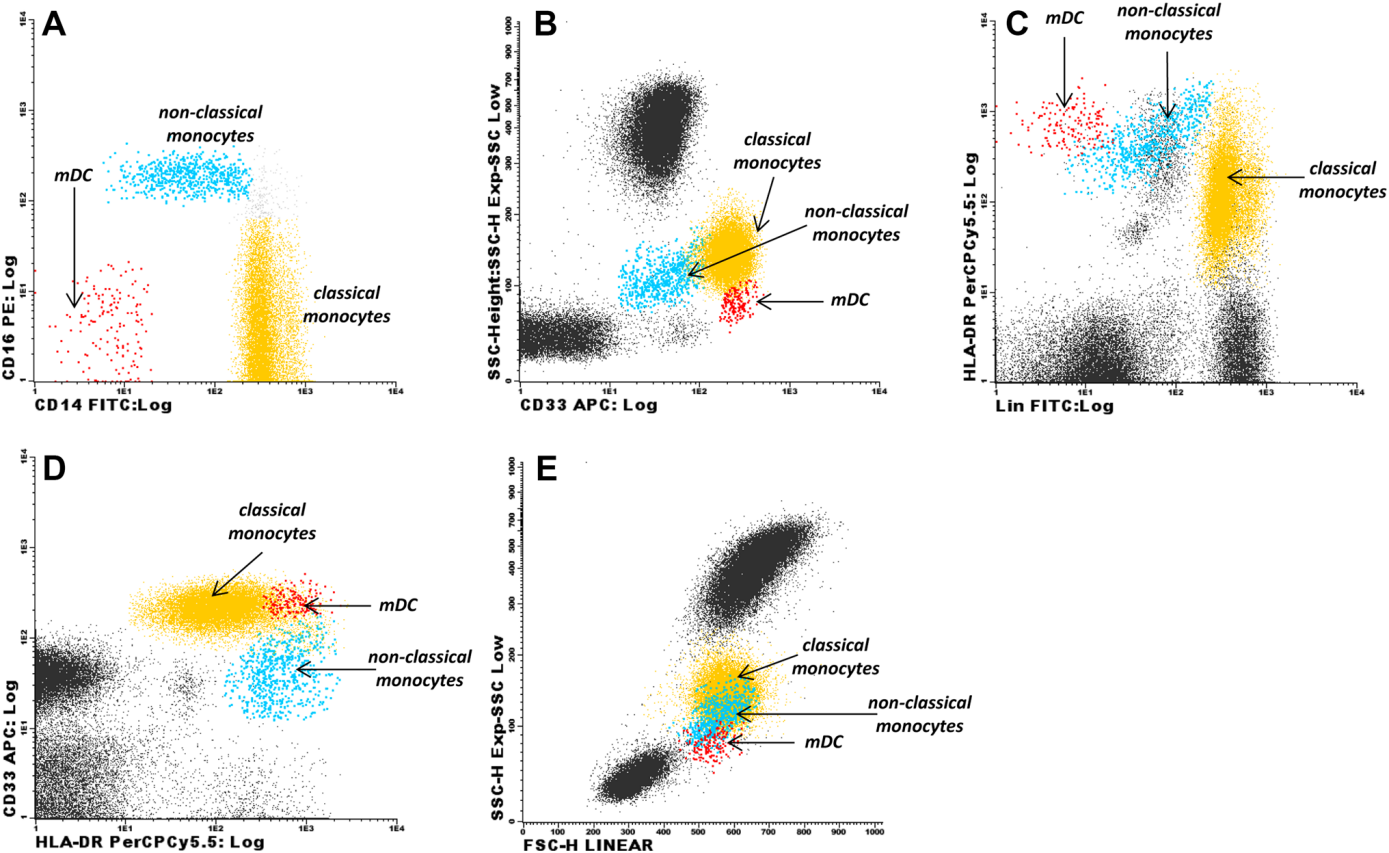
Flow cytometry strategy to identify classical, non-classical monocytes and myeloid dendritic cells (mDCs). **a** Conventional gating strategy is shown of classical and non-classical monocytes based on differential expression of CD14 and CD16: classical monocytes: CD14^++^CD16^−^ and non-classical monocytes: CD14^+/−^CD16^+^. **b, c** Since after LPS stimulation CD16 is downregulated, CD33 was used combined with CD14 and HLA-DR to distinguish the classical and non-classical monocytes: classical monocytes (CD33^++^, HLA-DR^+^, CD14^+^) is equivalent to CD14^++^CD16^−^; non-classical monocytes (CD33^low^, HLA-DR^inter^, CD14^low/−^) correspond to CD14^+^CD16^++^. **c, d** mDCs were identified based on the following phenotype: Lin^−^ (CD3^−^CD19^−^CD56^−^CD14^−^) CD33^++^HLA-DR^++^. **e** Monocytes and mDCs characteristics of forward scatter (FSC), and side scatter (SSC)

Considering that CD16 expression is lost shortly after LPS stimulation, as also reported by others [29, 30], preventing the identification of CD16 + monocyte subsets, while CD33 remains unchanged during LPS stimulation [30]; CD33 was used as an alternative marker to identify non-classical and classical monocytes [31]. Using CD16, CD14, HLA-DR and CD33 in unstimulated cells, it is possible to distinguish between non-classical and classical monocytes based on CD33 and CD14 combination (Fig. 1). mDCs were identified as lineage (CD3, CD19, CD56 and CD14)^neg^/HLA-DR^high^, CD33^high^ with intermediate forward and side scatter between lymphocytes and monocytes, as described already by others [32] (Fig. 1).

### Statistical analyses

Statistical evaluation of the data was performed using non-parametric tests, the associations between continuous variables were determined by Kruskal–Wallis test and Mann–Whitney *U* test and Chi-squared test for categorical variables. A Spearman’s rank correlation was applied to detect the association between different parameters. Two-sided testing was performed for all analyses. Differences and correlations were considered statistically significant at *p* < 0.05. Results were expressed as median and interquartile range. Statistical analyses were performed using Statistical Package for Social Sciences IBM SPSS 21 (IBM, Armonk, NY. USA) and Graphpad Prism version 6 (GraphPad Software, San Diego, CA, USA). Heatmaps and the unsupervised hierarchical clustering analysis were carried out using the Multiple Experiment Viewer (MeV) software, based on the Euclidean distance and the average linkage clustering.

## Results

### Patients with diffuse and limited cutaneous SSc display increased frequencies of cytokine secreting non-classical monocytes and myeloid dendritic cells

Classical, non-classical and intermediate monocyte subsets as well as mDC numbers in circulation, both frequency (%) and absolute values (cells/µL), were evaluated in SSc patients and HCs (see supplementary materials and methods). As depicted in supplementary Fig. S2, the percentage and absolute values of the intermediate monocyte subset, were found to be significantly increased in both lSSc and dSSc patients compared to HC. Additionally, significant higher absolute values of classical monocytes were observed in dSSc patients compared to the HC group.

Next the identification of cytokine production by classical, non-classical monocytes and mDCs was measured using the combination of CD33, HLA-DR and dendritic cell exclusion kit, as described by others and as represented in Fig. 1 [32, 33].

**Fig. 2:**
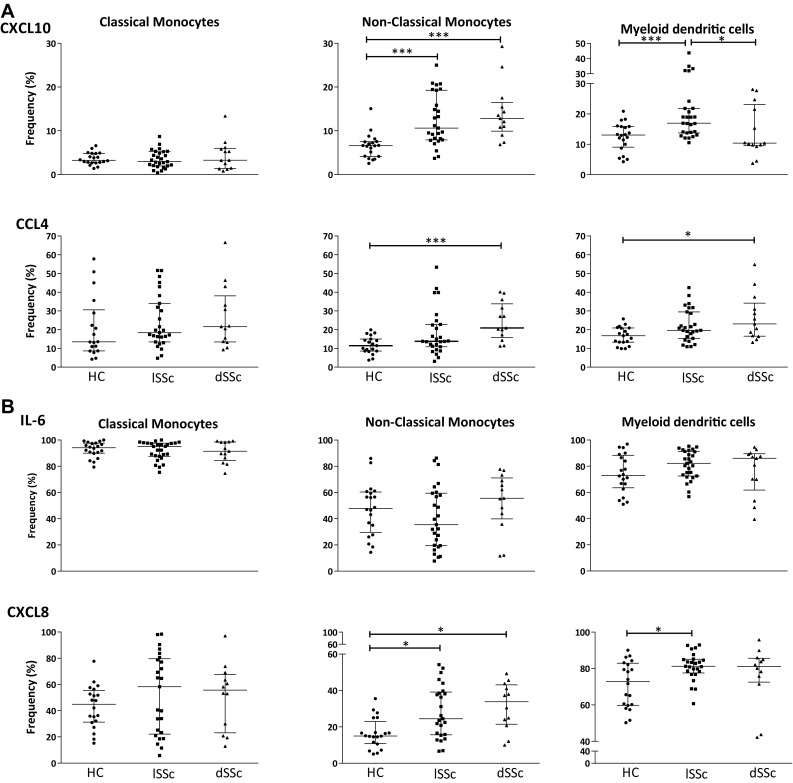
Non-classical monocytes and myeloid dendritic cells (mDCs) display a differentially enhanced ability of spontaneously produce CXCL10 and CCL4, as well as CXCL8 upon in vitro stimulation. **a** The frequency (%) of classical, non-classical monocytes and myeloid dendritic cells (mDCs) spontaneously producing CXCL10 and CCL4 is shown. **b** Frequency (%) of IL-6 and CXCL8-producing classical, non-classical monocytes and mDCs upon in vitro stimulation with LPS/IFN-γ. Statistically significant differences were considered when *p < 0.05 and ***p < 0.01 (Kruskal–Wallis followed by Mann–Whitney *U* test)

We evaluated the production of CXCL10, CCL4, CXCL8 and IL-6 in circulating classical monocytes, non-classical monocytes and myeloid DCs (mDCs) in patients with diffuse cutaneous (dSSc) and limited cutaneous (lSSc) SSc and compared this to that observed in healthy counterparts (representative dotplots are shown in supplementary Fig. S1). No differences were observed between the groups in the frequency of classical monocytes expressing CXCL10 or CCL4 (Fig. 2a). In contrast, frequencies of non-classical monocytes expressing CXCL10 were significantly increased in both patients with limited and diffuse cutaneous clinical SSc phenotype in comparison with healthy controls. CCL4-positive non-classical monocytes were more frequent only in patients with diffuse SSc. The percentage of mDCs expressing these chemokines was markedly different between the patient groups. CXCL10-expressing mDCs were more frequent in limited cutaneous SSc, while CCL4-expressing mDCs were increased only in individuals with diffuse SSc (Fig. 2a).

### SSc patients exhibit a higher frequency of non-classical monocytes and myeloid dendritic cells expressing CXCL8 after in vitro stimulation

As TLR and IFN-γ signalling are proven to be involved in SSc pathology and are well-known activators of chemokine production by monocytes and DCs, we next determined whether the stimulation by both LPS and IFN-γ resulted in a further enhanced difference in CCL4, CXCL10, CXCL8 or IL-6 expression by monocyte subsets and DCs between SSc subsets. The combination of LPS-IFN-γ led to a clear statistically significant increase in the expression of all four chemokines in all of these cell subsets (all *p* < 0.001, data not shown). This resulted in detectable frequencies of IL-6- and CXCL8-producing cells. Upon in vitro stimulation, however, the percentage of non-classical monocytes expressing CXCL8 was increased both in limited and diffuse SSc compared to HC. In addition, mDCs producing CXCL8 were statistically significantly more frequent in lSSc than in HC (Fig. 2b). For dSSc patients, a trend in CXCL8-secreting cells was observed.

In contrast to all other measured chemokines, the frequencies of IL-6-expressing cells were similar across all cell types and clinical subsets investigated (Fig. 2b).

### A cluster of CXCL10, CCL4, CXCL8 and IL-6- producing classical, non-classical monocytes and myeloid dendritic cells identifies a subgroup of SSc patients with lung fibrosis

**Fig. 3:**
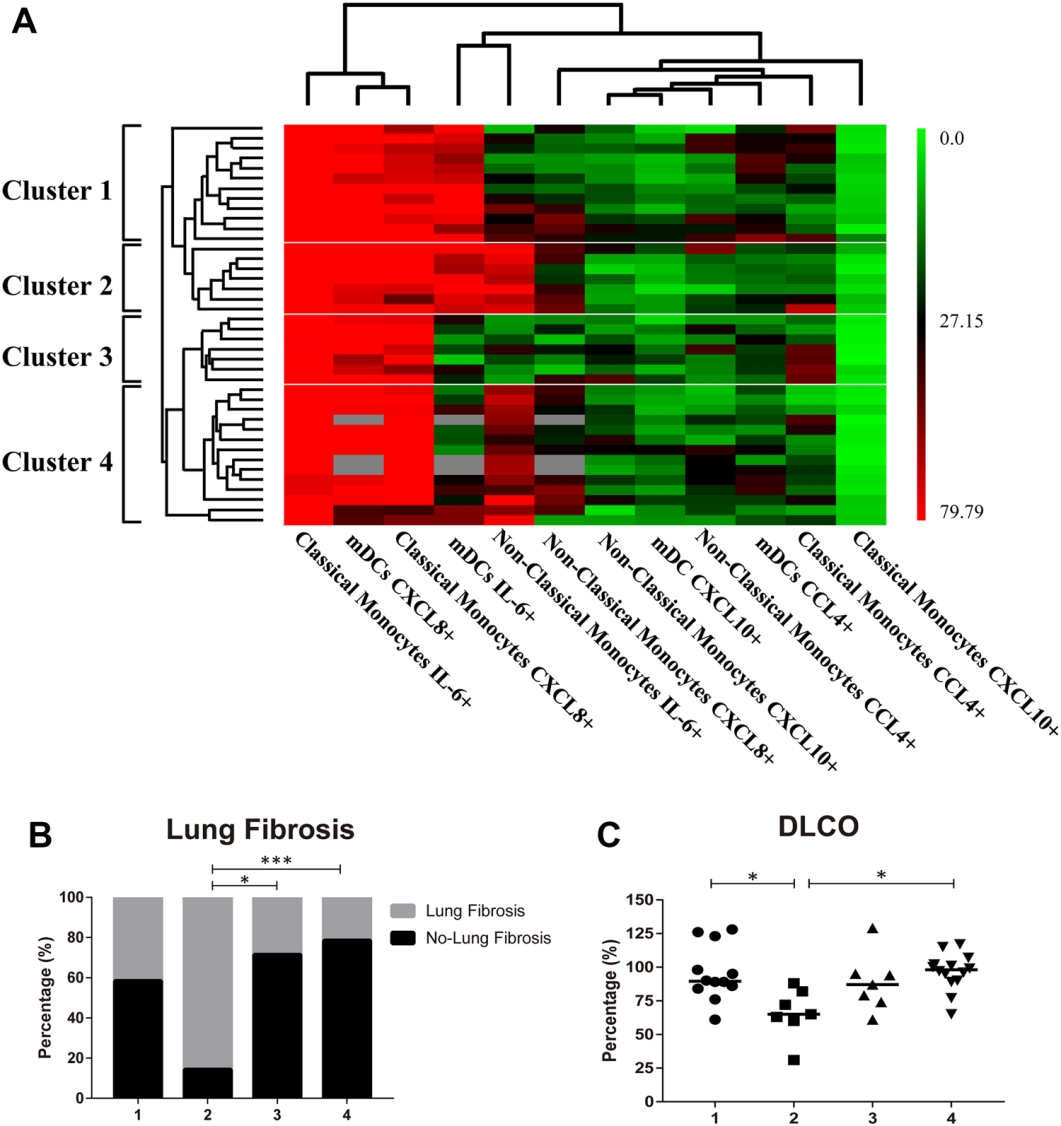
Unsupervised hierarchical clustering of cytokine secreting classical, non-classical monocytes and mDCs reveals a cluster that identifies a subgroup of SSc patients with increased lung fibrosis. **a** Heatmap colors represent the percentage of expressing cells in a color-coded way: green, lower expression compared to the mean; black, expression equal to the mean; red, higher expression compared to the mean; gray, missing values. Dendrograms indicating the clustering relationships are shown to the left and above the heatmap. **b, c** Cluster 2 reveals classical, non-classical monocytes and myeloid dendritic cells (mDCs) producing IL-6, CXCL8, CCL4 and CXCL10 that reflect SSc patients with lung involvement as witnessed by the presence of lung fibrosis or history of it (**b**) and DCLO measurements (**c**). Statistically significant differences were considered when *p < 0.05 and ***p < 0.01 (Chi-squared test for categorical variables and Kruskal–Wallis followed by Mann–Whitney *U* test for continuous variables). (Color figure online)

To evaluate a possible relation between the frequencies of classical, non-classical and mDC spontaneously producing CXCL10 and CCL4 as well as IL-6 and CXCL8 after in vitro stimulation and clinical characteristics of the SSc patients, an unsupervised hierarchical clustering was performed. This identified four distinct clusters (Fig. 3a). Comparison of clinical parameters between clusters shows that 85.7% of patients in cluster 2 present lung fibrosis (or history of); in addition, these patients also display a lower DLCO (diffusing capacity of the lung for carbon monoxide) (Fig. 3b). This cluster 2 was characterized by an increased frequency of classical monocytes expressing CXCL8, non-classical monocytes expressing IL-6 and CXCL8.

The presence of anti-nuclear or anti-centromere antibodies was not associated with frequencies of cells producing CXCL10, CCL4, CXCL8 or IL-6. In addition, no correlations of cytokine-producing cells with the mRss were observed (data not shown). Although clustering of all SSc patients in low and high mRSS (based on the score average of the SSc group) demonstrated a significant increased frequency of non-classical monocytes and mDCs expressing CCL4, which was not the case for low mRSS group, both groups were not statistically significantly different (Fig. S3). Also no significant differences were observed between patients that were or were not on corticosteroid therapy (Fig. S4).

## Discussion

SSc is a complex disease with heterogeneous clinical features and disease severity, corresponding to the extent of skin fibrosis and internal organ involvement, as a consequence of excessive collagen production and accumulation. It is believed that microvascular dysfunction may represent the early damage that incites acquired immune cells to respond to self-antigens finally leading to production of autoantibodies [34]. The importance of the innate immune system, in particular monocytes and DCs, and their ability to produce pro-inflammatory and pro-fibrotic cytokines and chemokines in SSc is increasingly recognized [7, 10, 23, 35]. In this study, we provide evidence for the contribution of classical, non-classical monocytes, but also Siglec 3-expressing mDC to SSc immunopathology.

Here, we show a higher frequency of circulating non-classical monocytes and mDCs that spontaneously produce CXCL10 in SSc. These data indicate that non-classical monocytes and mDCs could be an important source for CXCL10 production and therefore contribute to the increased serum levels of CXCL10 observed in SSc [13, 36]. Previous studies have demonstrated an association between high levels of CXCL10 and a more severe clinical phenotype, with lung and kidney involvement [8, 36]. In our study, we could not observe clear associations between the frequency of CXCL10-producing cells and disease activity markers, possibly indicating that other cell types contribute to increased CXCL10 levels. Alternatively, discrepancies between actually secreted and intracellular detected levels may contribute to the lack of correlation in this study.

CXCL10 is an IFN-responsive cytokine exerting its effects by binding to CXCR3, [37] mediating chemotaxis of T cells to the inflammation sites [38]. Although the role of CXCL10 in SSc is not completely understood, but its importance in the fibrotic process deserves attention. CXCL10 has been shown to act as a strong inhibitor of angiogenesis [39, 40] and has been implicated in the control of fibrosis in a mouse model of pulmonary fibrosis induced by bleomycin [41]. Remarkably, positive CXCL10 mDCs are particularly increased in lSSc, while positive CXCL10 non-classic monocytes in dSSc, which could represent a different role of these cell subsets in different disease stages. Numbers of CXCL10 cells might be dramatically affected by egress from bone marrow, activation and migration in different disease states.

In this study, we demonstrate increased numbers of non-classical monocytes and mDCs producing CCL4 in dSSc patients. CCL4 has been shown to act as a potent chemoattractant for monocytes and T cells [42], and serum levels and spontaneous production of CCL4 by peripheral blood mononuclear cells have been reported to be significantly elevated in patients with SSc [43–45]. Our data suggest that in particular non-classical monocytes might contribute to the enhanced CCL4 levels in SSc and potentially to fibrosis.

In patients with both limited and diffuse SSc, upon short-term stimulation with TLR4 ligand plus IFN-γ, increased numbers of CXCL8-secreting non-classical monocytes and myeloid dendritic cells were observed, indicating that these cells were probably primed in vivo. CXCL8 has been shown to promote angiogenesis and consequent fibrosis [46] and additionally the levels of CXCL8 in bronchoalveolar lavage fluid were found to be increased in SSc patients with interstitial lung disease [47] and in other fibrotic diseases, such as combined pulmonary fibrosis and emphysema [48]. This may indicate that the elevated levels of CXC chemokines, like CXCL8, in lungs contribute to fibrotic phenomena on going in SSc.

Here, we also demonstrate that CXCL10, CCL4, CXCL8 and IL-6-producing classical, non-classical monocytes and mDC profile identify a subset of patients characterized by the presence of lung fibrosis and decreased lung function. In these patients, CXCL8-expressing classical monocytes as well as non-classical monocytes producing IL-6 and CXCL8 are augmented. In line with these observations, serum IL-6 was previously found to be increased in patients with lung fibrosis and declined lung function [49, 50] and in patients with fibrosing alveolitis, an up-regulated CXCL8 secretion by alveolar macrophages was documented [51].

All together, our findings provide a novel evidence to suggest the contribution of activated circulating non-classical and classical monocytes and mDCs to the immunopathology of SSc. Our data suggest a complex interplay of different myeloid cell subsets, potentially contributing to both anti-fibrotic (CXCL10) and pro-fibrotic (CCL4, CXCL8) processes. Future studies should elucidate the dynamics and interplay of these subsets in the circulation and target organs to comprehend the relative contribution to the disease processes. The fact that current treatments (corticosteroids in this study) in SSc patients fail to inhibit cytokine secretion of the studied myeloid cells warrants further research to selectively target key pathogenic processes.

## Electronic supplementary material

Below is the link to the electronic supplementary material.

Supplementary material 1 (PDF 858 KB)

Supplementary material 2 (PDF 236 KB)
