# Supplementary material for: Increased frequencies of circulating CXCL10-, CXCL8- and CCL4-producing monocytes and Siglec-3-expressing myeloid dendritic cells in systemic sclerosis patients
Source: Inflamm Res. 2017 Nov 10;67(2):169–77. doi: 10.1007/s00011-017-1106-7 (PMC5765192; doi:10.1007/s00011-017-1106-7)

Inflammation Research

Increased frequencies of circulating CXCL10-, CXCL8- and CCL4-producing monocytes and Siglec-3-expressing myeloid dendritic cells in Systemic Sclerosis patients.

Tiago Carvalheiro[1,2,3*], Sara Horta[1,4*], Joel A.G. van Roon[2,3], Mariana Santiago[5], Maria J. Salvador[5], Hélder Trindade[1], Timothy R.D.J. Radstake[2,3], José A.P. da Silva[5,6], Artur Paiva[1,7]

Affiliations:

[1]Blood and Transplantation Center of Coimbra, Portuguese Institute of Blood and Transplantation, Coimbra, Portugal

[2]Department of Rheumatology & Clinical Immunology, University Medical Center Utrecht, the Netherlands.

[3]Laboratory of Translational Immunology, University Medical Center Utrecht, the Netherlands.

[4]Department of Chemistry, University of Aveiro, Aveiro, Portugal

[5]Department of Rheumatology, Coimbra University Hospital Center, Coimbra, Portugal,

[6]Faculty of Medicine, University of Coimbra, Coimbra, Portugal

[7]Flow Cytometry Unit, Clinical Pathology Service, Coimbra University Hospital Center, Coimbra, Portugal

*T. Carvalheiro and S. Horta equally contributed to this work.

Correspondent author:

Artur Paiva, *PhD*

Unidade de Gestão Operacional de Citometria, Serviço de Patologia Clínica

Praceta Prof. Mota Pinto, Ed. S. Jerónimo, 3º piso, 30001-301 Coimbra, Portugal

Tel: (+351) 239400400

E-mail: artur.paiva@chuc.min-saude.pt

Supplementary Fig. S1. Representative flow cytometry dot plots illustrating the quantification (%) of classical and non-classical monocytes and myeloid dendritic cells (mDC) A) spontaneously producing CXCL10 and CCL4 as well as B) IL-6 and CXCL8 upon in vitro stimulation with LPS/IFN-γ. One representative HC is depicted.

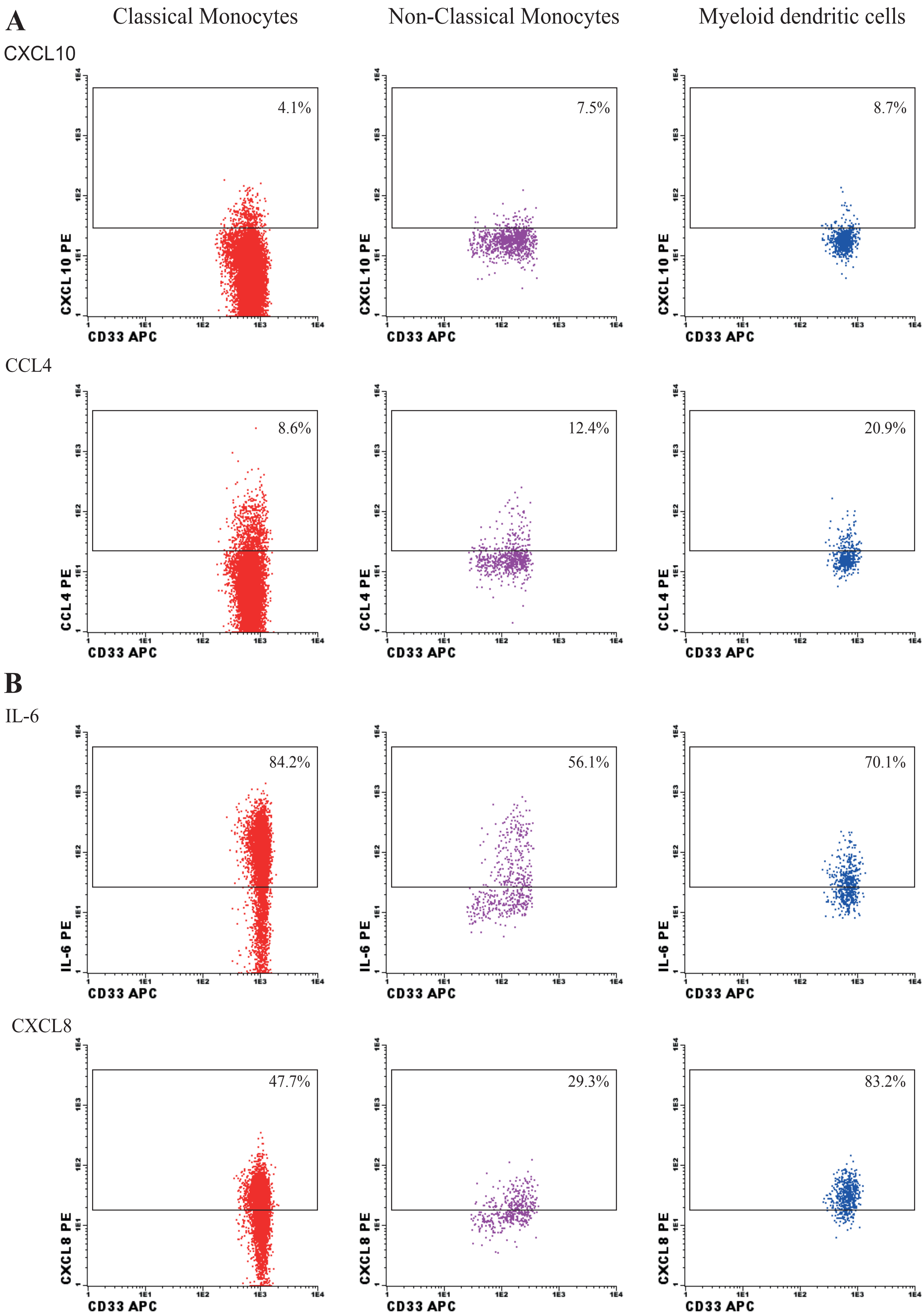

Supplementary Fig. S2. Quantification of classical, intermediate and non-classical monocytes subsets and myeloid dendritic cells (mDC) in SSc patients. A) Percentage within the CD45+ cells and B) absolute numbers indicated in number of cells *per* µL. Statistically significant differences were considered when *p<0.05 and *** p<0.01 (Kruskal-Wallis followed by Mann–Whitney U test).

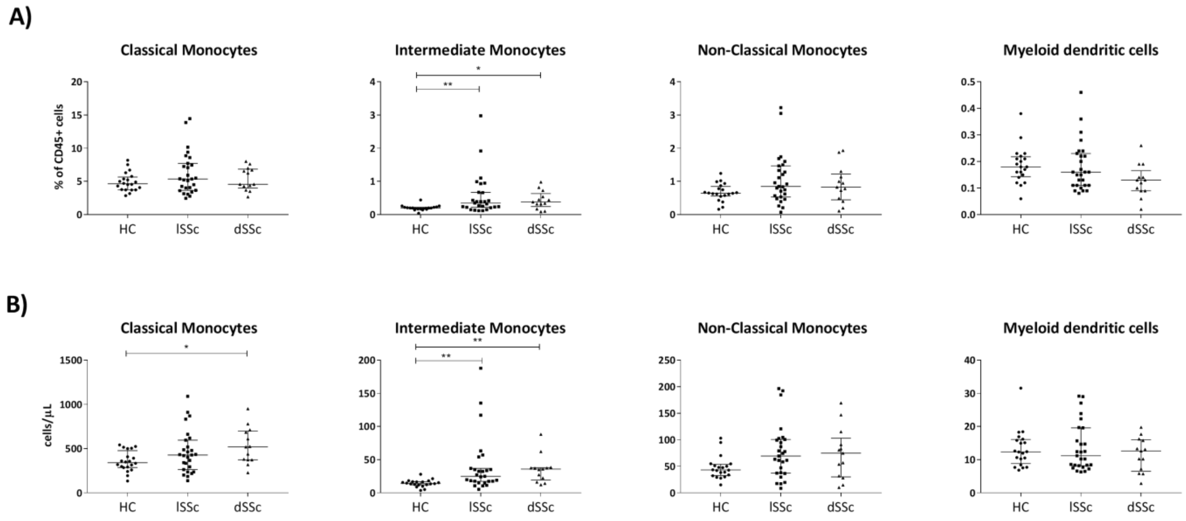

Supplementary Fig. S3. Frequency (%) of classical, non-classical monocytes and myeloid dendritic cells (mDCs) spontaneously producing CXCL10 and CCL4 cells and CXCL8 and IL-6 upon *in vitro* stimulation with LPS/IFN-γ, clustering SSc patients according to high and low mRss (base on the average of the score: 12). Statistically significant differences were considered when *p<0.05 and *** p<0.01 (Kruskal-Wallis followed by Mann–Whitney U test).

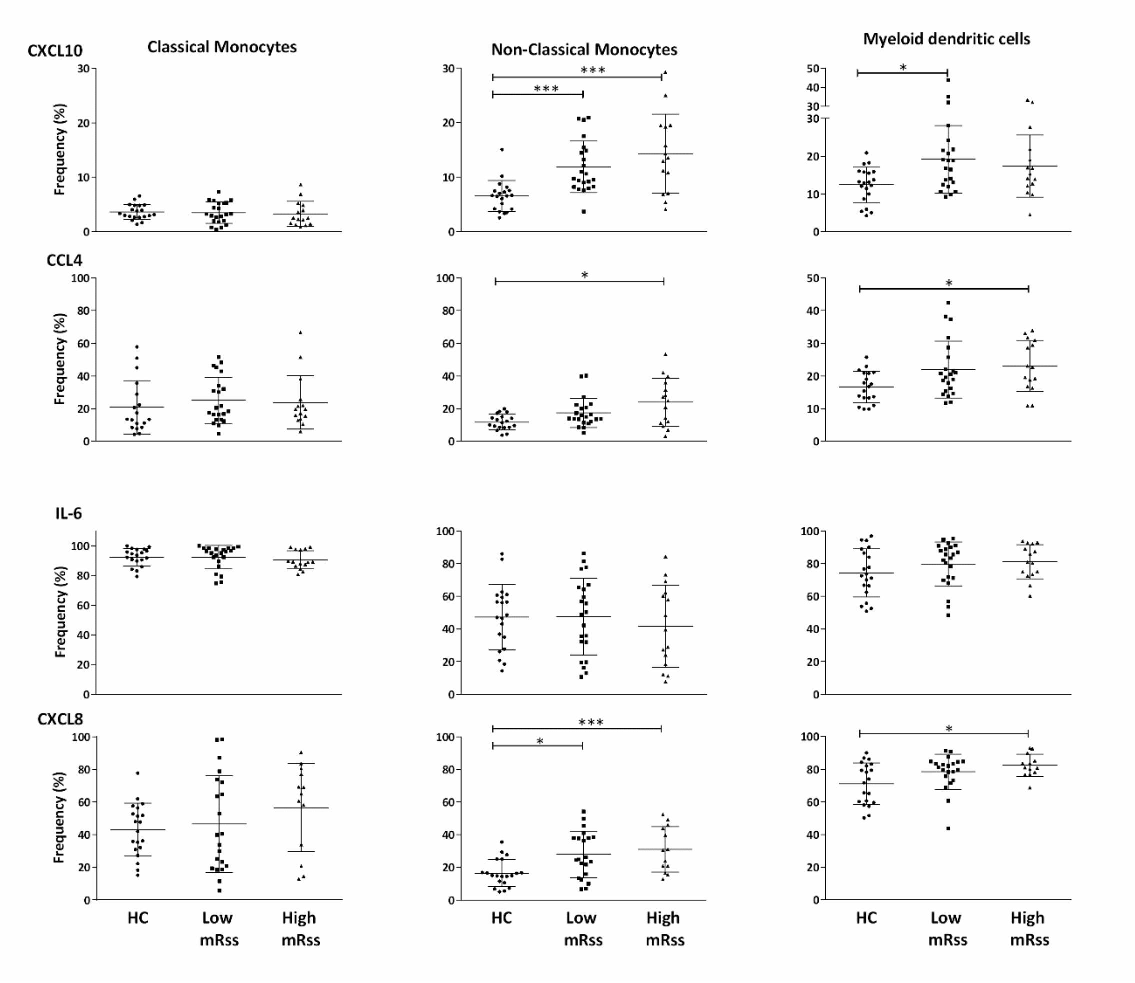

Supplementary Fig. S4. Frequency (%) of classical, non-classical monocytes and myeloid dendritic cells (mDCs) spontaneously producing CXCL10 and CCL4 cells and CXCL8 and IL-6 upon *in vitro* stimulation with LPS/IFN-γ, according to the treatment with corticosteroids.

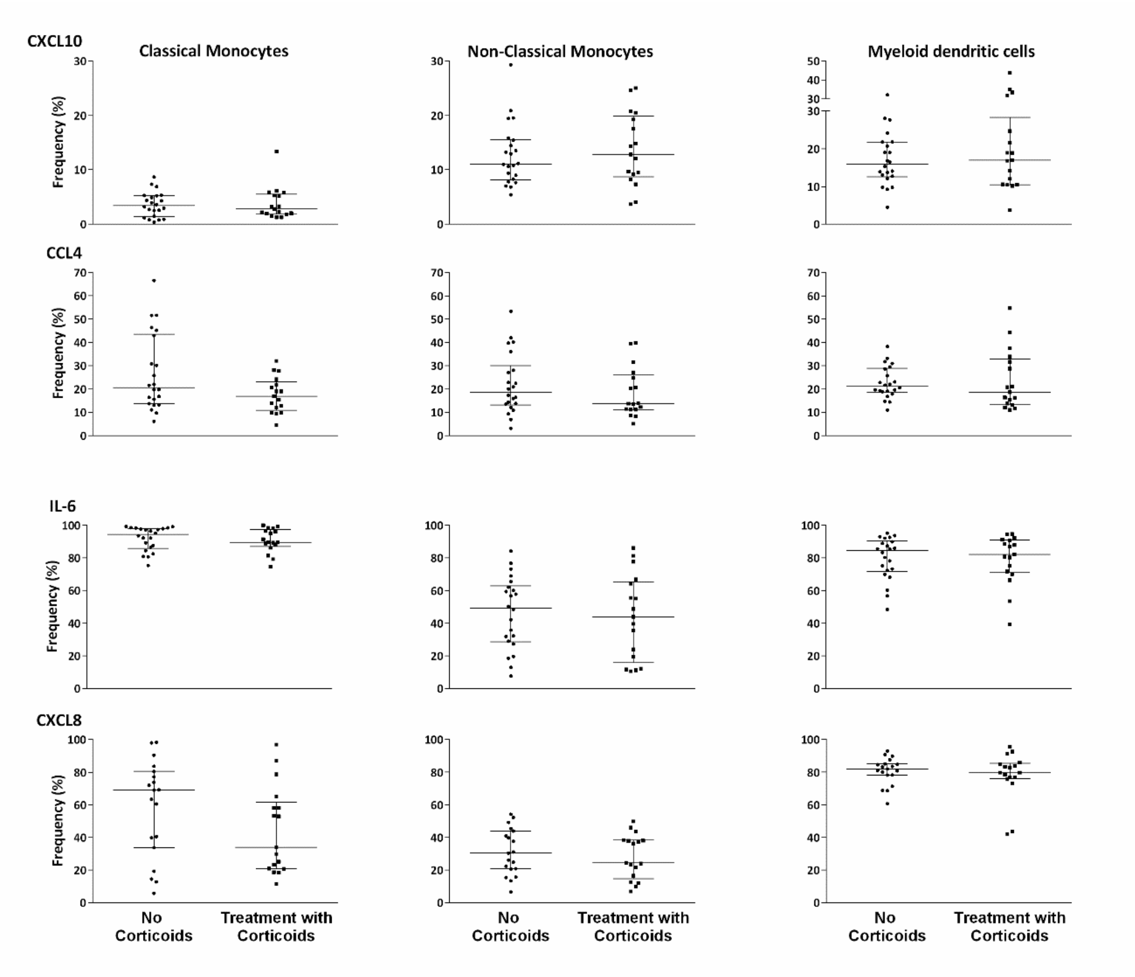

Supplement: Supplementary file 1 — Supplementary material 1 (PDF 858 KB) [file 11_2017_1106_MOESM1_ESM.pdf]
