# Supplementary material for: Increased frequencies of circulating CXCL10-, CXCL8- and CCL4-producing monocytes and Siglec-3-expressing myeloid dendritic cells in systemic sclerosis patients
Source: Inflamm Res. 2017 Nov 10;67(2):169–77. doi: 10.1007/s00011-017-1106-7 (PMC5765192; doi:10.1007/s00011-017-1106-7)
Supplement: Supplementary file 2 — Supplementary material 2 (PDF 236 KB) [file 11_2017_1106_MOESM2_ESM.pdf]

Inflammation Research

Increased frequencies of circulating CXCL10-, CXCL8- and CCL4-producing monocytes and Siglec-3-expressing myeloid dendritic cells in Systemic Sclerosis patients.

Tiago Carvalheiro[1,2,3*], Sara Horta[1,4*], Joel A.G. van Roon[2,3], Mariana Santiago[5], Maria J. Salvador[5], Hélder Trindade[1], Timothy R.D.J. Radstake[2,3], José A.P. da Silva[5,6], Artur Paiva[1,7]

Affiliations:

[1]Blood and Transplantation Center of Coimbra, Portuguese Institute of Blood and Transplantation, Coimbra, Portugal

[2]Department of Rheumatology & Clinical Immunology, University Medical Center Utrecht, the Netherlands.

[3]Laboratory of Translational Immunology, University Medical Center Utrecht, the Netherlands.

[4]Department of Chemistry, University of Aveiro, Aveiro, Portugal

[5]Department of Rheumatology, Coimbra University Hospital Center, Coimbra, Portugal,

[6]Faculty of Medicine, University of Coimbra, Coimbra, Portugal

[7]Flow Cytometry Unit, Clinical Pathology Service, Coimbra University Hospital Center, Coimbra, Portugal

*T. Carvalheiro and S. Horta equally contributed to this work.

CorrespondeSnt author:

Artur Paiva, *PhD*

Unidade de Gestão Operacional de Citometria, Serviço de Patologia Clínica

Praceta Prof. Mota Pinto, Ed. S. Jerónimo, 3º piso, 30001-301 Coimbra, Portugal

Tel: (+351) 239400400

E-mail: artur.paiva@chuc.min-saude.pt

Supplementary Materials and Methods

Quantification of monocytes subsets and myeloid dendritic cells

The quantification of classical, non-classical monocytes and myeloid dendritic cells (mDC) was performed in 0.2 mL of whole blood collected into K_3- Ethylenediamine tetraacetic acid (EDTA) tubes, stained with the following monoclonal antibodies combination: anti-CD45 Krome Orange (clone: J.33; Beckman Coulter – Immunotech; Marseille, France), anti-CD11c Phycoerythrin- Cyanine -5 tandem (clone: B-ly6, BD Pharmingen), anti-HLA-DR Pacific Blue (clone: Immu-357; Beckman Coulter – Immunotech), anti-CD14 Allophycocyanin- Hellite -7 tandem (clone: MφP9; BD Biosciences, San Jose, CA, USA), anti-CD16 Fluorescein Isothiocyanate (clone: CLB-Fc-gran/1; Sanquin, Amsterdam, the Netherlands), anti-CD33 Phycoerythrin- Cyanine -7 tandem (clone: D3HL60.251; Beckman Coulter – Immunotech). Sample were then incubated for 15 min at room temperature in darkness. After this incubation period, a lyse and wash protocol was followed: 2 mL of FACS Lysing Solution (BD Biosciences) diluted 1:10 (vol/vol) in distilled water was added, and after 10 min of incubation, the cells were washed with 2 mL of PBS and resuspended in 0.5 mL of PBS before acquisition.

Data acquisition was performed in an FACSCanto™II (BD Biosciences) flow cytometer equipped with FACSDiva software (version 6.1.2; BD Biosciences). For data analysis, Infinicyt (version 1.6) software (Cytognos SL, Salamanca, Spain) was used. Absolute counts were calculated using a dual platform methodology (flow cytometry and hematological cell analyzer).
